# Effects of Mobile App–Based Intervention for Depression in Middle-Aged and Older Adults: Mixed Methods Feasibility Study

**DOI:** 10.2196/25808

**Published:** 2021-06-29

**Authors:** Christine E Gould, Chalise Carlson, Flora Ma, Valerie Forman-Hoffman, Kristian Ranta, Eric Kuhn

**Affiliations:** 1 Geriatric Research, Education, and Clinical Center VA Palo Alto Health Care System Palo Alto, CA United States; 2 Department of Psychiatry and Behavioral Sciences Stanford University Palo Alto, CA United States; 3 Pacific Graduate School of Psychology Palo Alto University Palo Alto, CA United States; 4 Meru Health, Inc San Mateo, CA United States; 5 National Center for Posttraumatic Stress Disorder VA Palo Alto Health Care System Menlo Park, CA United States

**Keywords:** aging, depression, digital health, digital therapeutics, mHealth, mobile phone

## Abstract

**Background:**

Digital mental health interventions may help middle-aged and older adults with depression overcome barriers to accessing traditional care, but few studies have investigated their use in this population.

**Objective:**

This pilot study examines the feasibility, acceptability, and potential efficacy of the Meru Health Program, an 8-week mobile app–delivered intervention.

**Methods:**

A total of 20 community-dwelling middle-aged and older adults (age: mean 61.7 years, SD 11.3) with elevated depressive symptoms participated in a single-arm pilot study investigating the Meru Health Program, an app-delivered intervention supported by remote therapists. The program primarily uses mindfulness and cognitive behavioral skills to target depressive symptoms. A semistructured interview was completed at the baseline to establish current psychiatric diagnoses. Depressive symptoms were measured using the Patient Health Questionnaire and Patient-Reported Outcomes Measurement Information System (PROMIS) depression measures. Anxiety symptoms were measured using the Generalized Anxiety Disorder Scale and the PROMIS Anxiety measure. User experience and acceptability were examined through surveys and qualitative interviews.

**Results:**

In total, 90% (18/20) of the participants completed the program, with 75% (15/20) completing at least 7 of the 8 introductory weekly lessons. On average, participants completed 60 minutes of practice and exchanged 5 messages with their therapists every week. The app was rated as helpful by 89% (17/19) participants. Significant decreases in depressive (P=.03) and anxiety symptom measures (P=.01) were found; 45% (9/20) of participants showed clinically significant improvement in either depressive symptoms or anxiety symptoms.

**Conclusions:**

The findings suggest that the commercially available Meru Health Program may be feasible, acceptable, and potentially beneficial to middle-aged and older adults. Although larger controlled trials are needed to demonstrate efficacy, these findings suggest that digital health interventions may benefit adults of all ages.

## Introduction

### Background

Major depressive disorder (MDD) is among the foremost causes of disability worldwide among older adults [1-3]. In adults aged 55 years and older in the United States, 5.6% experienced MDD within a 1-year period [4]. Two nonpharmacological interventions, cognitive behavioral therapy (CBT) and mindfulness-based interventions have been efficacious in reducing late-life depressive symptoms [5-7]. However, high rates of depression persist because of undertreatment [8,9].

Older adults face multiple barriers in accessing mental health interventions, including limited transportation and mobility, living distally from treatment settings, and high health care costs, which could be addressed with digital health interventions [10,11]. As smartphone use grows, interest in using mobile apps to deliver therapeutic interventions has proliferated [12]. A meta-analysis [13] of randomized controlled trials of mobile apps for depression in adults found a moderate effect size than in inactive controls and a small effect size than in active controls. The applicability of these findings to older adults is unknown because the majority (65%) of randomized controlled trials in the meta-analysis included younger participants (age: mean ≤30 years). Trends in smartphone ownership suggest that older adults, particularly baby boomers, are rapidly adopting mobile devices [14], yet many older adults have not yet used mental health–related mobile apps, despite reported interest and openness to these interventions [15,16].

Few studies have examined mental health–related mobile apps among older users. One group tested an adapted app-based intervention to promote the self-management of chronic conditions alongside peer coaching among middle-aged and older adults with serious mental illness and found it to be feasible, acceptable, and potentially helpful in improving outcomes among the 8 study completers [17]. Another small study examining an app targeting mental wellness in a group-based treatment setting yielded positive feedback about the app from older users, but the findings were limited by substantial attrition [18]. These small studies suggest the acceptability of mental health apps among older users when used alongside in-person support (ie, peer coaches and group-based support). Given the barriers to care faced by older adults, there is a need to examine app-based interventions that do not include extensive in-person support.

### Objectives

This study investigates the use of an 8-week mobile app–based intervention with remote therapist support called the Meru Health Program (version 2.0), a commercially available digital health program. The Meru app contains video instructional content and guided practices delivered to a group of 5 to 10 individuals who work through the program as a cohort. Videos and practices teach CBT and mindfulness techniques to manage depressive and related symptoms, such as worry. Patients have access to a dedicated therapist via asynchronous within-app messaging or, if needed, a phone call. The therapist provides guidance by (1) responding to patients’ messages, (2) sending unprompted messages to patients and encouraging program adherence, and (3) reviewing responses to practice exercises. The app also includes a moderated discussion where group members can share thoughts and experiences with practices and support other patients by providing limited feedback on their posts.

An uncontrolled study of the Meru Health Program in young adults demonstrated feasibility and reduced depressive symptoms [19]. Similarly, a second uncontrolled study found that 60% exhibited clinically significant improvement in depressive symptoms at the 12-month follow-up [20]. This study extends to an understudied age group of middle-aged and older adults. It examined the feasibility, acceptability, and preliminary efficacy of the Meru Health Program among middle-aged and older adults using a mixed methods approach.

## Methods

### Participants

Participants were recruited through various announcements describing a mobile app intervention for depression using (1) flyers posted at medical offices and on community boards (eg, at libraries and coffee shops), (2) web-based advertisements (Craigslist and Facebook), (3) word of mouth, and (4) invitations to previous research participants interested in future research opportunities.

The inclusion and exclusion criteria were assessed during the telephone screening. Inclusion criteria were age ≥40 years, elevated depressive symptoms, owning a smartphone, and residing in California because of telehealth laws regarding practicing across state lines. Exclusion criteria included cognitive impairment, suicidal ideation, current participation in psychotherapy, problematic drinking, and psychotic symptoms. Participants taking psychotropic medications must have been on a stable dose (>1 month) to be included.

### Measures

#### Demographic and Health Questionnaire

Demographic information and health status, presence of eight major health conditions (arthritis, asthma or bronchitis, cancer, diabetes, epilepsy, heart disease, hypertension, and stroke), and vision and hearing impairment were gathered at baseline via questionnaires.

#### The Mobile Device Proficiency Questionnaire

The Mobile Device Proficiency Questionnaire (MDPQ) [21] examines experience with and aptitude for using mobile devices across eight domains: mobile device basics, communication, data and file storage, internet, calendar, entertainment, privacy, troubleshooting, and software management. Each of the 46 items is rated using a 5-point scale with an option for “never tried” (1) and then ratings for ease of completion from “not at all” (2) to “very easily” (5). The total score ranges from 8 to 40, with higher scores reflecting greater proficiency. Internal consistency was excellent (Cronbach α=.96). The MDPQ was completed at the baseline.

#### Patient Health Questionnaire 9-Item

The Patient Health Questionnaire 9-item (PHQ-9) [22] measures the frequency of depression symptoms over the last 2 weeks. Total scores range from 0 to 27, with higher scores reflecting increased depressive symptom severity. Internal consistency varied across timepoints (baseline α=.60; week 5 α=.82; posttreatment α=.77). The PHQ-9 was completed at the telephone screen, baseline, week 5, and week 8 (posttreatment). Within the app, the PHQ-9 was completed during weeks 1, 3, 5, 7, and 8.

#### Generalized Anxiety Disorder 7-Item Scale

The Generalized Anxiety Disorder 7-item (GAD-7) scale [23] measures anxiety symptoms characterizing Generalized Anxiety Disorder. Total scores range from 0 to 21, with higher scores reflecting increased anxiety symptom severity. Internal consistency was acceptable to good across timepoints (baseline α=.77; week 5 α=.83; posttreatment α=.86). The GAD-7 was completed at baseline, week 5, and posttreatment. Within the app, the GAD-7 was completed during weeks 1, 5, and 8.

#### Patient-Reported Outcomes Measurement Information System Depression and Anxiety Measures

The Patient-Reported Outcomes Measurement Information System (PROMIS) Depression (short form 8a) and Anxiety (short form 8) scales were used as secondary measures [24]. Total scores ranged from 8 to 40 on the PROMIS Depression and from 7 to 35 on the PROMIS Anxiety, with higher scores reflecting increased symptom severity. The PROMIS Depression and Anxiety scores had good-to-excellent internal consistency at baseline (α=.90 and .89, respectively) and posttreatment (α=.97 and .95, respectively). The PROMIS measures were completed at baseline and at weeks 5 and 8.

#### Mini International Neuropsychiatric Interview

The Mini International Neuropsychiatric Interview (MINI 7.0.2) [25] is a semistructured interview that assesses psychiatric disorders using the Diagnostic and Statistical Manual of Mental Disorders 5th Edition [26] criteria. The MINI psychotic screen was administered during the telephone screen; the remainder was administered at baseline to assess current major depression and other comorbid psychiatric disorders. For diagnostic accuracy and supervision purposes, the principal investigator conducted live or audio-recorded observations for half of the interviews conducted by the research staff.

#### App Use

The app use data collected included the number of messages sent, duration of use, lessons completed, and the number and types of practices completed.

#### User Experience Survey

At posttreatment, participants completed a 6-item self-report measure about their experiences and impressions of the program (Multimedia Appendix 1). Participants ranked the four components of the app (information, practices, therapist chat, and group support) from most to least helpful. Participants then responded to five statements about the ease of use of the app, helpfulness of the app, helpfulness of the emails, frequency of the emails, and duration of the program using a 5-point Likert-type scale ranging from 1 (strongly disagree) to 5 (strongly agree).

#### Qualitative Interview

After treatment, participants completed a semistructured qualitative interview about the program (Multimedia Appendix 1). They were asked about helpful and unhelpful aspects of the program, technical or navigational issues, program benefits, and suggested improvements. The interviews were audio-recorded, transcribed, and reviewed for transcription accuracy.

### Procedures

#### Initial Screening and Baseline Assessment

The study was approved by the Stanford University School of Medicine Institutional Review Board and registered at ClinicalTrials.gov (NCT03652948). The study was conducted between July 2018 and May 2019.

Potential participants completed a telephone screen to assess the inclusion and exclusion criteria. The PHQ-9 [22] determined the presence of elevated depressive symptoms (≥10) [27]. If PHQ-9 item 9 (death or suicide ideation) was endorsed as *several days* or more, then the P4 Suicide Risk Screener [28] was administered to assess active suicidal ideation and, if present, individuals were excluded and referred for treatment. Questions about current mental health treatment assessed for current psychotherapy and use of medications. The Short Blessed Test [29] identified possible cognitive impairments (≥6). The Alcohol Use Disorders Identification Test (AUDIT-C) [30] was used to identify problematic drinking (≥5). Finally, possible psychotic disorders were identified using a psychotic screen from the MINI [25].

Eligible participants were invited to an in-person visit, during which the research staff obtained informed consent, followed by administration of the complete MINI and self-report assessments. Participants then received a handout explaining the steps to begin the Meru Health Program. First, study referrals were sent directly to the Meru therapist, who scheduled an introductory call with the participant. Five days before the start of the program, participants received an email with instructions on downloading and logging into the app on their own smartphone.

#### Meru Health Program

**Figure 1:**
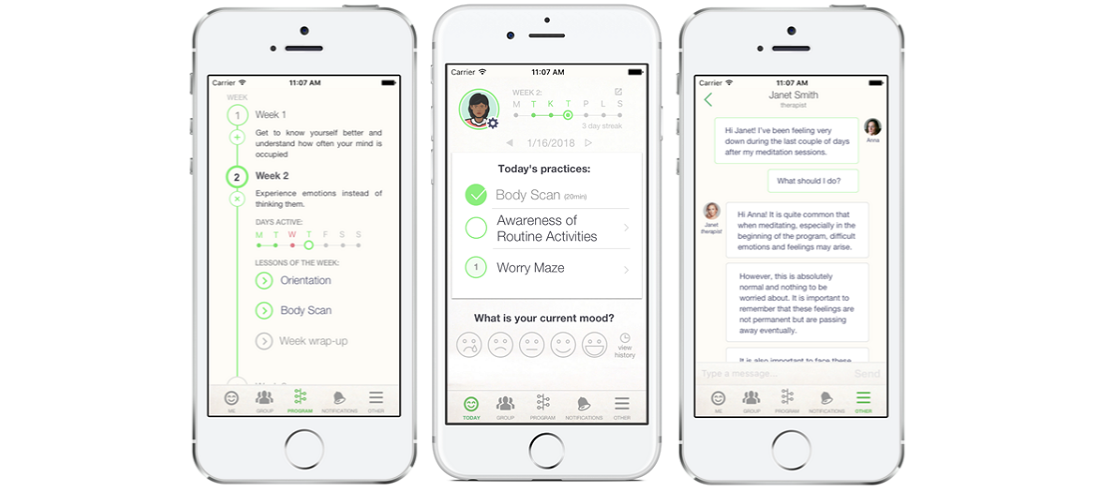
Meru Health Program version 2.0 screenshots.

Meru Health is a registered health care provider in multiple states in the United States and Finland. Individuals primarily access the Meru Health Program by selecting health and employee plans. The app is available for download via the App Store (iOS) or Play Store (Android), but patients require a referral and eligibility screen to access the app after download. As described earlier, the program consists of an app, therapist support (via app and weekly emails), and group discussion. The app has five menu options: (1) “today screen” with informational videos and practices to be completed, (2) group discussion page, (3) program timeline, (4) notifications, and (5) asynchronous within-app messaging with the therapist (Figure 1). Participants can review previous days’ content through the program timeline screen and are advised of missed content through notifications. The interactive aspects of the program include the group-based discussion feature wherein participants can share their thoughts, and other members can respond with either a heart icon or one of four supportive statements.

Meru Health therapists are licensed practitioners with doctoral or master’s degrees in psychology, counseling, social work, marriage and family therapy, or other related fields. Therapists have formal training in mindfulness, CBT, and manual training (>30 hours), which includes an assessment of skill acquisition to deliver the Meru Health Program. Therapists send weekly emails introducing new content (ie, CBT or mindfulness skills) to be learned within the app each week. Therapists monitor practice completion; send messages to encourage adherence; and review participants’ messages and group discussion posts for possible suicide or homicidal ideation, active substance use, or the presence of psychotic symptoms. The therapist responded to participants exhibiting these symptoms or those who had evidence of worsening mental health within 12 hours. In addition, posts deemed inappropriate or potentially harmful to other group members (ie, triggering) were removed.

#### 5- and 8-Week (Posttreatment) Assessments

Participants were contacted by researchers during week 5 to complete questionnaires assessing depressive and anxiety symptoms. At the posttreatment assessment, participants completed these questionnaires again and provided feedback about the program via a user experience survey and qualitative interview. Participants received payment only for completion of assessments administered by the research team at baseline, week 5, and week 8/posttreatment (total compensation=US $80; US $40 for baseline and posttreatment assessment). No other payment was provided.

### Statistical Analysis

To assess the feasibility of the program, we summarized participant flow, inspected retention rates, and calculated the mean use of different app components using the app use data. Acceptability was examined by summarizing responses to the user experience survey.

The qualitative interview transcripts were excerpted by the first author (CEG) using Dedoose 8.2.14 (SocioCultural Research Consultants) web-based software [31]. A codebook was developed using inductive (ie, review of transcripts) and deductive methods. Excerpts were independently coded by the 2 coders. The codebook was revised and adjudicated three times to reach a prespecified reliability of *κ*>0.70 (final pooled *κ*=0.81). Excerpts were discussed among 3 authors (CEG, CC, and FM), and matrices were created to examine variations in patterns or themes by quantitative ratings consistent with a mixed methods approach [32].

Intervention effects on depressive and anxiety symptoms were estimated using linear mixed effects models using SPSS version 24 (IBM) [33]. All participants were included in the analyses, which used intention-to-treat principles. The models included random intercepts and slopes and used residual maximum likelihood estimation owing to the small sample size [34]; 5% of the data points on dependent variables were missing because of dropouts. Improvement in symptoms indicative of response was defined as having equal to or surpassing the minimally clinically important difference of five points or more [35] on the PHQ-9 or 4 points or more [36] on the GAD-7.

### Data Availability

A limited data set that supports the findings of this study is available from the corresponding author upon reasonable request.

## Results

### Participant Characteristics

Participants (N=20) had a mean age of 61.65 years (SD 11.32; range 42-81 years), and most were women (14/20, 70%) and White and non-Hispanic (12/20, 60%; Table 1). In total, 60% (12/20) participants were aged 60 years or older; 70% (14/20) met the criteria for the current MDD.

**Table 1:** Participant characteristics (N=20).

Participant characteristics			Values
Age (years), mean (SD)			61.65 (11.32)
Education (years), mean (SD)			16.60 (2.46)
**Sex, n (%)**			
	Female	14 (70)	
	Male	6 (30)	
**Race and ethnicity, n (%)**			
	White, non-Hispanic	12 (60)	
	Any race, Hispanic	2 (10)	
	Asian	3 (15)	
	Multiracial	3 (15)	
**Marital status, n (%)**			
	Single	10 (50)	
	Married	3 (15)	
	Separated or divorced	6 (30)	
	Widowed	1 (5)	
**Current living situation^a^, n (%)**			
	Alone	9 (45)	
	With spouse or partner	7 (35)	
	With relative or roommate	3 (15)	
**Employment, n (%)**			
	Full-time	6 (30)	
	Part-time	2 (10)	
	Unemployed	7 (35)	
	Retired	5 (25)	
Taking psychotropic medications, n (%)			10 (50)
**Self-rated health, n (%)**			
	Excellent	1 (5)	
	Good	11 (55)	
	Fair	6 (30)	
	Poor	2 (10)	
**Current medical conditions, n (%)**			
	Arthritis	10 (50)	
	Asthma or bronchitis	7 (35)	
	Cancer	1 (5)	
	Diabetes	2 (10)	
	Heart disease	2 (10)	
	Hypertension	7 (35)	
**Sensory difficulties, n (%)**			
	Use eyeglasses	18 (90)	
	Hearing loss (both ears)	7 (35)	
	Use hearing aids	3 (15)	
**Prevalence of current psychiatric diagnoses (MINI^b^), n (%)**			
	Major depressive disorder	14 (70)	
	Anxiety disorders	12 (60)	
	Posttraumatic stress disorder	3 (15)	
	Other disorders^c^	5 (20)	
MDPQ^d^, mean (SD)			34.68 (4.62)

^a^N=19. Percentages of medical conditions, sensory difficulties, and psychiatric diagnoses do not add up to 100% because participants may have had more than 1 condition.

^b^MINI: Mini International Neuropsychiatric Interview.

^c^Other disorders include alcohol use (early remission), obsessive-compulsive disorder, and binge eating disorder.

^d^MDPQ: Mobile Device Proficiency Questionnaire.

### Feasibility, User Experience, and Acceptability

**Figure 2:**
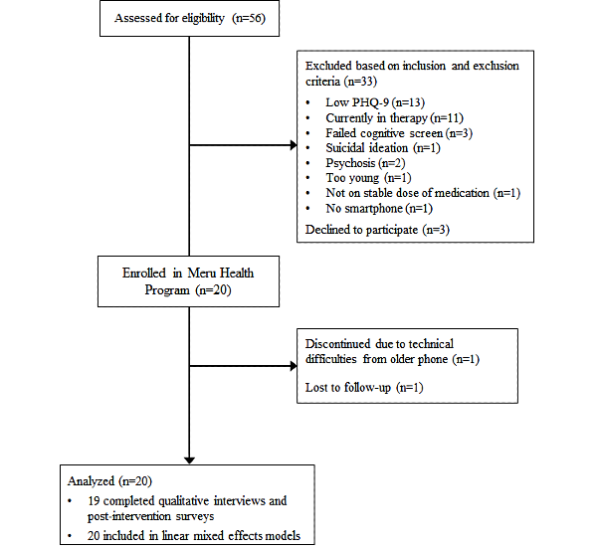
Flow of participants through the study. PHQ-9: Patient Health Questionnaire 9-item.

As displayed in Figure 2, 90% (18/20) participants completed the program, and 2 discontinued the program. One individual (aged >60 years) discontinued the program because of technical challenges with using the app on an older smartphone but completed the postassessment; the other (aged <60 years) discontinued the program because of spontaneous symptom improvement and activities conflicting with app use and did not complete the postassessment. Of the 4 groups conducted during the study, 3 included some Meru Health Program nonstudy patients; one group (n=5) included only study participants. The groups’ sizes ranged from 5 to 9 participants. In the mixed groups of study and nonstudy participants, the nonstudy patients comprised half (3/6, 50%) of the participants in one group or a smaller minority (1/9, 11% and 2/6, 33%) in the other two groups. Each group was led by 1 of 2 therapists. No qualitative differences emerged based on which therapist led the groups or whether the groups consisted of study participants only or study and nonstudy participants. Per the app use data (N=20), all participants downloaded the app, logged on, messaged with their therapist, and practiced at least once. The mean duration of time spent on practices throughout the program was 9.07 hours (SD 6.67), which averaged out to approximately 68 minutes each week. Participants sent 13.4 (SD 10.89) messages to their therapist, with a range spanning 2 to 38 messages. Most (15/20, 75%) completed at least 7 of the 8 weekly introductory lessons.

The user experience survey results displayed in Table 2 highlight that the program was deemed usable by most with some nuances identified during the qualitative interview. Most participants (15/19, 79%) indicated that the program somewhat or completely met their expectations, and 89% (17/19) believed the app was helpful. Examinations of the core program components revealed that the practices were deemed most helpful, and group discussion was the least helpful (Table 3). The inclusion of a therapist proved very important, as highlighted by one participant:

Having the therapist there for me was great in the fact that I felt like it wasn’t just a computer that I was interacting with in the program. She provided a personal touch which was very important in the program. Because I think we all, at least me, like to be validated by a human, it makes it [the program] more real to me...I had her in case the app was bothering me, I needed a connection to someone that I can talk to about, or if I didn’t understand something, I had her to be there for me.

Another program component, the group discussion component, was deemed the least helpful. However, some participants appreciated reading what others posted (Table 2).

**Table 2:** User experience survey and qualitative interview findings.

Item	Responses, n (%)	Qualitative interview findings	Example quotes
App usability: the Meru Health app was easy to use.^a^	5 (25): strongly agree; 12 (60): agree 0 (0): neutral 2 (10): disagree 0 (0): strongly disagree	Challenges included difficulty reviewing previous content, typing long answers in response to questions after practices, progress not being saved, crashing, or freezing.	“On some of them [practices] I could pause and rewind and play again, but some of them I could only pause and play.” “I’m a typer as opposed to a tapper. I’m much more able to ramble like I’d like to if I had a keyboard in front of me...I seem to have a better hand at expressing my thoughts when I can go back and quickly edit.”
Communication: the emails from Meru Health were helpful to me.^b^	9 (45): strongly agree 4 (20): agree 4 (20): neutral 1 (5): disagree 0 (0): strongly disagree	Some expected more communication directly with the therapist. Three were unaware that they would be receiving emails.	“I occasionally got confused whether messages would show up in my email inbox or whether they would show up on the app.” “[emails] would break down the topics, and I think that was fabulous, that was very informative. Without that information, it would have been difficult because it was not really explained, I went back and read the emails a few times, so it would make some sense.”
Communication frequency: the emails from Meru Health were frequent enough.^b^	11 (55): strongly agree 6 (30): agree 0 (0): neutral 1 (5): disagree 0 (0): strongly disagree	Weekly emails were frequent enough for most participants. Most comments reflected a desire for more personalized communication.	“I thought there would be a little more of the individual therapist or individual communication between the therapist and myself, just a little more of that, a lot more...”
Program length: the Meru Health Program was the right length of time.^a^	9 (45): strongly agree 4 (20): agree 2 (10): neutral 3 (15): disagree 1 (5): strongly disagree	Six participants recommended that it could be longer (9 to 24 weeks). One recommended it be shorter (4 weeks).	“I think it would be great if the app gives [the] participant a time to choose from, for me 3 months would have been ideal.”

^a^n=19.

^b^n=18.

**Table 3:** Mixed methods evaluation of program components (n=19).

Component	Most helpful^a^, n (%)	Least helpful, n (%)	Helpful aspects	Areas to improve
Practices	12 (63)	0 (0)	Having narrator-guided practices Specific practices, spanning CBT^b^ and mindfulness deemed helpful Included 3-minute reset, breathing, mindfulness, self-compassion, establishing boundaries, and thought record	Usability issues such as loss of progress if interrupted (or exiting app) while practicing Desire for chimes or signals when practices end (too much silence) Need for more introductory practices for certain components (eg, self-compassion) Clearer option to skip reflection questions after practices
Therapist Support	7 (37)	2 (11)	Therapist was caring, thoughtful, genuine, and supportive Provided helpful feedback and comments on participant entries Helped personalize the program by providing additional information and resources when necessary	Unclear how frequently to interact with therapist Unclear how much information to share with therapist
Information Provided	4 (21)	2 (11)	Education and information about mood and thinking patterns Provides underlying rationale for CBT and mindfulness practices	One participant requested practices to help differentiate rumination from reflection concerning thought boundaries.
Group	0 (0)	15 (79)	Reading others’ responses helped people feel less alone and feel validated in their struggles.	Confusion about how to use the group and need for guidelines Low rates of participants using group Limited response options (preprogrammed drop-down) to other members’ comments

^a^Three individuals ranked more than 1 component as most helpful; 1 person did not select the most helpful component.

^b^CBT: cognitive behavioral therapy.

Qualitative findings highlighted the aspects of each component that were confusing or would benefit from improvement. Specifically, participants wanted more guidance about how best to use aspects of the program, ranging from questions about the interface to the need for some guidelines in using the therapist and group support. Regarding group support, participants expected to have more flexibility in responding to other group members:

A couple of things I read that other people [posted], I wanted to give a comment on, but you were limited to 4 choices...it didn’t give you the opportunity to say anything personal, positive, in your own words.

Other suggestions pertained to having an improved orientation to the app and guidance on how frequently they communicate with the therapist.

### Preliminary Effects of the Program on Mental Health Symptoms

**Figure 3:**
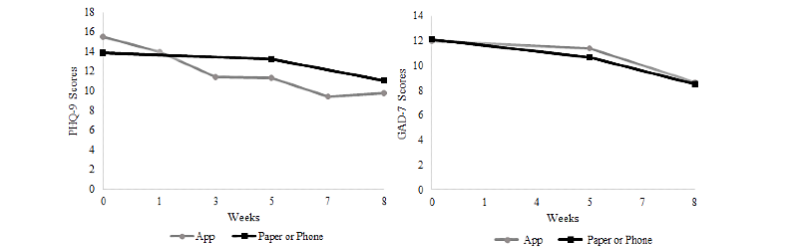
Mean depression and anxiety symptoms measured within the app and by paper or phone assessments. GAD-7: Generalized Anxiety Disorder 7-item; PHQ-9: Patient Health Questionnaire 9-item.

Figure 3 displays the mean PHQ-9 and GAD-7 mean scores across timepoints. Table 4 presents the results of the linear mixed effects model. Time was significant (P=.03-.001) in all 4 linear mixed models, which demonstrated that both depressive and anxiety symptoms reduced across the 8-week program (Table 4). The (uncontrolled) effect sizes ranged from medium to large (Hedges *g* values=.41-.94). Regarding clinically significant improvement, 30% (6/20) participants had improvements in depressive symptoms and 45% (9/20) in anxiety symptoms. All 6 who experienced improvement in depression also showed improvement in anxiety. Age and mobile device proficiency were not significantly associated with change in scores on any outcome measures, but older age was associated with lower mobile device proficiency (*r*=−0.60; P=.005). Mobile device proficiency total scores ranged from 26.47 to 40, which falls above the average for older adults (mean 19.2, SD 10.5) in the initial MDPQ validation paper [21].

**Table 4:** Mean assessment scores across timepoints and linear mixed effects models.

Measure	Baseline, mean (SD)	5 weeks, mean (SD)	8 weeks^a^, mean (SD)	Parameter estimate (SE)	*t* value (*df*)	P value	Hedges g
PHQ-9^b^	13.90 (4.25)	12.95 (5.86)	10.95 (5.77)	−1.36 (0.58)	−2.33 (19.63)	.03	0.53
GAD-7^c^	12.10 (4.24)	10.33 (5.42)	8.53 (5.18)	−1.74 (0.65)	−2.67 (23.09)	.01	0.69
PROMIS Depression	29.05 (6.96)	24.89 (7.64)	21.21 (8.18)	−3.84 (1.03)	−3.72 (20.32)	<.001	0.94
PROMIS Anxiety	25.25 (6.47)	23.11 (5.92)	21.32 (10.32)	−2.01 (0.83)	−2.40 (20.88)	.03	0.41

^a^N=19 for week 8. Parameter estimates for main effects (time) correspond to changes across each time point (baseline, midpoint, and end of program).

^b^PHQ-9: Patient Health Questionnaire 9-item.

^c^GAD-7: Generalized Anxiety Disorder 7-item.

## Discussion

### Principal Findings

Our findings suggest the feasibility and acceptability of an 8-week mobile app–based intervention for depressive and anxiety symptoms in a sample of middle-aged and older adults approximately 30 years older than samples in most mobile intervention research. The low dropout rate (10%) and robust engagement with the app mirrors findings with the Meru Health Program in younger adults [19,20] and with an average dropout rate of 17% for internet or mobile interventions [37]. Similarly, the high satisfaction with the app, good ratings of usability, and overall perception that the program was helpful by most participants underscores the acceptability of this intervention in older age groups.

Older adults with depression may benefit from mobile interventions, such as the Meru Health Program, based on significant reductions in depression and anxiety outcomes. This and the finding that 45% (9/20) of individuals achieved a clinically significant reduction in symptoms suggest that the program may be beneficial to some middle-aged and older users. The uncontrolled effect sizes were slightly larger than, but comparable with, those found in a meta-analysis of mobile interventions for depression [13]. Larger controlled studies are needed to replicate our findings and to explore the moderators of treatment outcomes, such as cognition in older adults with depressive symptoms.

The qualitative findings provide an important context to help elucidate specific aspects of the feasibility and acceptability of the program. As expected, the practices were found to be the most helpful component, which is consistent with previous findings regarding guided self-management programs in older adults [38]. The therapist was a critical aspect of the program, particularly for participants who desired human contact, support, and encouragement or struggled with the practices or the app itself. The support model mostly used *pull* support where users reach out for help but also incorporated some *push*-based support (eg, feedback on practice reflections or weekly messages). According to the Efficiency Model of Support, the limitations of pull support include underutilization because of concerns about burdening the supporter [39]. Our findings corroborate this with participants describing uncertainty about how often to communicate with the therapist and warrant further consideration for program refinement.

Group support was a unique, yet underutilized, program component. Being part of a group facing similar struggles was helpful to some in promoting understanding and a sense of not being alone. Unfortunately, the limited response options for communicating with other members undermined the use of and interest in this feature. Thus, this component should be considered for modification to increase its utilization. Such future enhancements will need to balance the risks of providing open-ended interactions, including potential breaches in confidentiality.

Another potential refinement to the program that might especially benefit older adults is an enhanced program orientation, including an in-person or preview of the app, a handout, instructional videos, or a combination of these. Such an orientation may encourage greater exploration of the app, as older users tend to be hesitant about making errors or pressing incorrect buttons, resulting in less app exploration compared with younger users. Other suggestions for program improvements included greater control over video-delivered information (eg, ability to rewind videos to review content). Program compatibility with tablets could help older users experience dexterity and vision challenges. Including older users in usability testing and research on digital health interventions would ensure that these interventions are optimized for all users. Furthermore, ensuring universal design is an important step before dissemination in health care settings, such as primary care that serves patients across the life span.

### Limitations

Conclusions from our findings must consider the limitations of the study. Foremost among these was the lack of a control condition, leaving open the question of whether the intervention was responsible for symptom improvement. Second, most of our sample had at least 16 years of education, which may limit the generalizability of the findings to those with less education. Third, our sample for the pilot study was quite small, with 90% (18/20) of participants completing the intervention. Although the sample included 40% (8/20) non-White individuals, because of the small overall sample size, further research is needed to evaluate the efficacy of this intervention in larger and even more racially and ethnically diverse samples. Fourth, a standardized mobile app rating scale or evidence-based usability measure was not used to assess the acceptability or usability of the Meru Health app. Fifth, mobile device proficiency for the sample was substantially higher than that evidenced in the initial study of the MDPQ [21]; thus, the findings may not generalize to individuals less proficient with mobile devices.

### Conclusions

In summary, this study found evidence of feasibility and acceptability of a commercially available app, the Meru Health Program, in a small sample of middle-aged and older users. Furthermore, the findings show that some participants experienced reduced psychiatric symptoms. Although larger, controlled trials are needed to demonstrate efficacy, our findings align with research with younger adults and suggest that digital health interventions may benefit and should be offered to adults of all ages. If future research validates these preliminary findings, mobile mental health interventions could be a valuable tool in addressing unmet treatment needs and reducing the burden of depression among older adults.

## Appendix

Multimedia Appendix 1 Meru Health Program study user experience and interview.

## Abbreviations

AUDIT-C: Alcohol Use Disorders Identification Test
CBT: cognitive behavioral therapy
GAD-7: Generalized Anxiety Disorder 7-item
MDD: major depressive disorder
MDPQ: Mobile Device Proficiency Questionnaire
MINI: Mini International Neuropsychiatric Interview
PHQ-9: Patient Health Questionnaire 9-item
PROMIS: Patient-Reported Outcomes Measurement Information System
