# Supplementary material for: Effects of Mobile App–Based Intervention for Depression in Middle-Aged and Older Adults: Mixed Methods Feasibility Study
Source: JMIR Form Res. 2021 Jun 29;5(6):e25808. doi: 10.2196/25808 (PMC8278301; doi:10.2196/25808)
Supplement: Multimedia Appendix 1 [file formative_v5i6e25808_app1.doc]

**Multimedia Appendix 1.** Meru Health Program study user experience and interview.

**Meru Health Program Feedback *Survey***

Please rank the helpfulness of each part of the Meru Health Program from 1 (most helpful) to 4 (least helpful):

**____ Information provided ____ Practices**

**____ Chat with therapist ____ Group support**

Instructions: Please circle your response below each of the following statements.

1. The Meru Health app was easy to use.

| 1 | 2 | 3 | 4 | 5 |
| --- | --- | --- | --- | --- |
| Strongly Disagree | Disagree | Neutral | Agree | Strongly Agree |

1. The Meru Health app was helpful to me.

| 1 | 2 | 3 | 4 | 5 |
| --- | --- | --- | --- | --- |
| Strongly Disagree | Disagree | Neutral | Agree | Strongly Agree |

1. The emails from Meru Health were helpful to me.

| 1 | 2 | 3 | 4 | 5 |
| --- | --- | --- | --- | --- |
| Strongly Disagree | Disagree | Neutral | Agree | Strongly Agree |

1. The emails from Meru Health about the program were frequent enough.

| 1 | 2 | 3 | 4 | 5 |
| --- | --- | --- | --- | --- |
| Strongly Disagree | Disagree | Neutral | Agree | Strongly Agree |

If you did not circle a 5 in question 4 above, how often would you like to be have been called about the Meru Health Program to check in?___________________________________

______________________________________________________________________________

1. The Meru Health Program was the right length of time.

| 1 | 2 | 3 | 4 | 5 |
| --- | --- | --- | --- | --- |
| Strongly Disagree | Disagree | Neutral | Agree | Strongly Agree |

If you did not circle a 5 above in question 5 above, how long would you have liked the program to be? ________________________________________________________________________

_____________________________________________________________________________

**Meru Health Program** **Feedback *Interview***

*The interview portion of the assessment is intended as a Semi-Structured Guide. Interviewers may ask follow up prompts to clarify participant answers.*

*Treatment Expectations and Impressions*

1. *How did you expect that your depression symptoms would be treated in this program?
2. *How well did this program meet your expectations?

*Please rate using below scale (ratings provided on visual aid)*

| Completely met my expectations | Somewhat met my expectations | Somewhat did not meet my expectations | Did not meet my expectations at all |
| --- | --- | --- | --- |

*Follow-up*: Can you tell me more about how the program did or did not meet your expectations?

1. *What about this program was most helpful?
   1. *What was not helpful?
   2. * What was difficult?
   3. Did you encounter problems using the app? [If so, please describe.]
2. How could we improve the support (emails, etc) that we provide to help you use the Meru Health Program Coach?
3. How specifically did you use the Meru Health Program during the study?
4. What specific concerns did it [insert helpful aspect of app] help?
5. What suggestions do you have for improving the app?

*Functioning and Well-being*

1. *What effects, if any, did you notice from this program?

*Follow-up questions*

1. How, if at all, did the treatment affect your well-being?
2. How, if at all, did the treatment affect the things you do or don’t do? [GOAL: identify any effects on activities and functioning]
3. [*If the program was helpful* *to participant*] How soon after the program began did you notice improvement?

Week 2 when the daily practices started to sink in, and let therapist in.

Note. *Indicates question is from Dakin and Arean (2013) *AJGP* article. Questions 1 and 2 are similar to the article, but the phrasing has been modified to lower the reading level (to 8^th^ grade) and improve clarity.
